# Co-occurrence and distribution of East (L1014S) and West (L1014F) African knock-down resistance in *Anopheles gambiae sensu lato* population of Tanzania

**DOI:** 10.1111/tmi.12248

**Published:** 2014-01-06

**Authors:** Bilali Kabula, William Kisinza, Patrick Tungu, Chacha Ndege, Benard Batengana, Douglas Kollo, Robert Malima, Jessica Kafuko, Mahdi Mohamed, Stephen Magesa

**Affiliations:** 1 National Institute for Medical Research, Amani Research CentreMuheza, Tanzania; 2 Kilimanjaro Christian Medical University College, Tumaini UniversityMoshi, Tanzania; 3 National Institute for Medical Research, Mwanza Research CentreMwanza, Tanzania; 4 The Presidents' Malaria Initiative, PMI/USAID OfficeDar es Salaam, Tanzania; 5 Global Health Division, RTI InternationalDar es Salaam, Tanzania; 6 Global Health Division, RTI InternationalNairobi, Kenya

**Keywords:** kdr, L1014S, L1014F, insecticide resistance, *Anopheles gambiae*, Tanzania

## Abstract

**Objective:**

Insecticide resistance molecular markers can provide sensitive indicators of resistance development in Anopheles vector populations. Assaying these makers is of paramount importance in the resistance monitoring programme. We investigated the presence and distribution of knock-down resistance (*kdr)* mutations in *Anopheles gambiae s.l*. in Tanzania.

**Methods:**

Indoor-resting Anopheles mosquitoes were collected from 10 sites and tested for insecticide resistance using the standard WHO protocol. Polymerase chain reaction-based molecular diagnostics were used to genotype mosquitoes and detect *kdr* mutations.

**Results:**

The *An. gambiae* tested were resistance to lambdacyhalothrin in Muheza, Arumeru and Muleba. Out of 350 *An. gambiae s.l*. genotyped, 35% were *An. gambiae s.s*. and 65% *An. arabiensis*. L1014S and L1014F mutations were detected in both *An. gambiae s.s*. and *An. arabiensis*. L1014S point mutation was found at the allelic frequency of 4–33%, while L1014F was at the allelic frequency 6–41%. The L1014S mutation was much associated with *An. gambiae s.s*. (χ^2^ = 23.41; *P *<* *0.0001) and L1014F associated with *An. arabiensis* (χ^2^ = 11.21; *P *=* *0.0008). The occurrence of the L1014S allele was significantly associated with lambdacyhalothrin resistance mosquitoes (Fisher exact *P *<* *0.001).

**Conclusion:**

The observed co-occurrence of L1014S and L1014F mutations coupled with reports of insecticide resistance in the country suggest that pyrethroid resistance is becoming a widespread phenomenon among our malaria vector populations. The presence of L1014F mutation in this East African mosquito population indicates the spreading of this gene across Africa. The potential operational implications of these findings on malaria control need further exploration.

**Objectif:**

Les marqueurs moléculaires de la résistance aux insecticides peuvent fournir des indicateurs sensibles du développement de la résistance dans les populations de vecteurs *Anopheles*. Le test de ces indicateurs est d'une importance énorme dans le programme de surveillance de la résistance. Nous avons étudié la présence et la répartition des mutations de résistance knockdown (kdr) chez *Anopheles gambiae* s.l. en Tanzanie.

**Méthodes:**

Des anophèles d'intérieur, au repos ont été collectées dans 10 sites et testées pour la résistance aux insecticides en utilisant le protocole standard de l'OMS. Les diagnostics moléculaires basés sur la PCR ont été utilisés pour le génotypage des moustiques et la détection des génotypes *kdr*.

**Résultats:**

Les *An. gambiae* testées étaient résistantes à la lambdacyhalothrine à Muheza, Arumeru et Muleba. Sur 350 *An. gambiae* s.l. génotypées, 35% étaient *An. gambiae* s.s. et 65% étaient *An. arabiensis*. Les mutations L1014S et L1014F ont été détectées à la fois chez *An. gambiae* s.s. et *An. arabiensis*. La mutation ponctuelle L1014S a été trouvée à la fréquence allélique de 4 à 33%, tandis que L1014F était à la fréquence allélique de 6 à 14%. La mutation L1014S a été fortement associée à *An. gambiae* s.s. (Chi carré = 23,41; *P*<0,0001) et L1014F était associée à *An. arabiensis* (chi carré = 11,21; *P* = 0,0008). L'allèle L1014S était significativement associé aux moustiques résistants à la lambdacyhalothrine (Fisher *P* exact <0,001).

**Conclusion:**

La cooccurrence des mutations L1014S et L1014F couplées à des rapports sur la résistance aux insecticides suggèrent que la résistance aux pyréthrinoïdes est en train de devenir un phénomène répandu dans les populations de vecteurs du paludisme en Tanzanie. La présence de la mutation L1014F dans cette population de moustiques en Afrique de l'Est indique la propagation de ce gène à travers l'Afrique. L'investigation des implications opérationnelles potentielles de ces résultats sur le contrôle du paludisme devraient être approfondie.

**Objetivo:**

Los marcadores moleculares de resistencia a insecticidas pueden ser indicadores sensibles del desarrollo de resistencias en las poblaciones de los vectores *Anopheles*. Evaluar dichos marcadores es crucial para los programas de monitorización de resistencias. Hemos investigado la presencia y la distribución de las mutaciones de resistencia *knockdown (kdr)* en *Anopheles gambiae s.l*. en Tanzania.

**Métodos:**

Se recolectaron mosquitos Anopheles intradomiciliarios de 10 lugares diferentes y se evaluaron en busca de resistencia a insecticidas utilizando el protocolo estándar de la OMS. Mediante un diagnóstico molecular basado en la PCR se genotiparon los mosquitos y se detectaron los genotipos *kdr*.

**Resultados:**

Los *An. gambiae* evaluados eran resistentes a lambdacialotrina en Muheza, Arumeru y Muleba. De 350 *An. gambiae s.l*. genotipados, 35% eran *An. gambiae s.s*. y 65% eran *An. arabiensis*. Se detectaron mutaciones L1014S y L1014F tanto en *An. gambiae s.s*. como en *An. arabiensis*. La mutación puntual L1014S se encontró con una frecuencia alélica de 4-33%, mientras que L1014F tenía una frecuencia alélica de 6-14%. La mutación L1014S estaba ampliamente asociada a *An. gambiae s.s*. (Chi-Cuadrado = 23.41; *P* < 0.0001) y la L1014F estaba asociada con *An. arabiensis* (Chi-Square = 11.21; *P* = 0.0008). El alelo L1014S estaba significativamente asociado con mosquitos resistentes a la lambdacialotrina (*P* < 0.001).

**Conclusión:**

La simultaneidad de mutaciones de L1014S y L1014F junto con informes de resistencia a los insecticidas sugiere que la resistencia a piretroides se está convirtiendo en un fenómeno común entre las poblaciones del vector de la malaria en Tanzania. La presencia de la mutación L1014F en estas poblaciones del Este de África indican la diseminación del gen a lo largo del continente africano. Determinar las implicaciones potenciales a nivel operativo de estos hallazgos sobre el control de la malaria requiere de más estudios.

## Introduction

Malaria vector control programmes in Africa rely heavily on the use of pesticides for insecticide-treated nets (ITNs)/long-lasting insecticide-treated nets (LLINs) and for indoor residual spraying (IRS)(WHO 2012b). The use of these strategies is known to contribute in the reduction in malaria transmission (Lengeler 2002; Pluess *et al*. 2010). The effectiveness of the current vector control depends much on the susceptibility of the local malaria vectors to insecticides used (WHO 2012a). Four major classes of chemical insecticides (i.e. pyrethroids, organochlorines, organophosphates and carbamates) are the mainstay of these malaria vector control strategies (Najera & Zaim 2002; WHO 2006; Kelly-Hope *et al*. 2008). All of these four classes are recommended for IRS. Pyrethroids are the only class of insecticide currently recommended for use on ITNs/LLINs because of their irritant and fast-acting properties and their safety for humans (Zaim *et al*. 2000). These major classes of chemical insecticides are nerve poisons and either target acetylcholinesterase in the synapses or the voltage-gated sodium channel in the insect neurones. Pyrethroids and DDT are neurotoxins that act on the voltage-gated sodium channels by modifying their gating kinetics, resulting in the prolonged opening of individual channels leading to paralysis and death of the insect (Ranson *et al*. 2011).

Massive use of insecticides in agriculture (Yadouleton *et al*. 2010) and public health (Czeher *et al*. 2008; Trape *et al*. 2011) has resulted in increasing resistance among malaria vectors due to the selection pressure placed on resistance genes (Ranson *et al*. 2011). Reduced susceptibility of Anopheles mosquitoes to insecticides such as DDT (dichloro-diphenyl-trichloroethane), malathion, fenitrothion, propoxur and bendiocarb was first reported in 1950s (Brown 1958; Hamon *et al*. 1968). To date, resistance among Anopheles species to at least one of the four commonly used insecticide classes has been reported in 64 malaria-endemic countries worldwide, the vast majority reporting resistance to pyrethroids (WHO 2012a,2012b). Even in four insecticide classes available for IRS, resistance has been reported for all of them in some populations of *Anopheles gambiae s.s* (Ranson *et al*. 2009). The increasing resistance of malaria vectors to available insecticides especially pyrethroids, puts current global control efforts at risk.

The major mechanisms by which insects acquire resistance to insecticides are elevated levels of detoxifying enzymes (metabolic resistance) and target-site insensitivity (Hemingway & Ranson 2000; Ranson *et al*. 2011). Metabolic resistance to pyrethroids is mostly associated with increased cytochrome P450 activity (Berge *et al*. 1998; Vulule *et al*. 1999). Recent studies have reported overexpression of cytochrome P450 genes: CYP6M2, CYP6P3 and CYP6Z2 in pyrethroid-resistant populations of *An. gambiae* (Muller *et al*. 2007, 2008; Djouaka *et al*. 2008; Mitchell *et al*. 2012).

Target-site insensitivity in *An. gambiae* is associated with two distinct mutations in the S6 transmembrane segment of domain II of the para-type sodium channel at position 1014. The mutations result in either a leucine–phenylalanine (L1014F) (Martinez-Torres *et al*. 1998) or a leucine–serine (L1014S) substitution (Ranson *et al*. 2000). The former mutation, which leads to the substitution of a leucine (TTA) for phenylalanine (TTT), was first detected in populations of the Savanna chromosomal form and S molecular form of *An. gambiae* s.s. in coastal Ivory Coast (Elissa *et al*. 1993). This was later found to be widespread in West Africa and reported to be strongly associated with pyrethroid resistance in *An. gambiae* (Martinez-Torres *et al*. 1998; Chandre *et al*. 1999a). The latter *kdr* mutation, with the same amino acid substituting the leucine (TTA) for serine (TCA), was first described in East African *An. gambiae s.s*. (Ranson *et al*. 2000). Both types of *kdr* mutations have been linked with DDT and pyrethroid-resistant phenotypes in wild *An. gambiae s.l*. populations (Martinez-Torres *et al*. 1998; Kolaczinski *et al*. 2000; Ranson *et al*. 2000; Donnelly *et al*. 2009).

Several studies with limited geographical sampling have attempted to detail the distribution of *kdr* mutations in *An. gambiae*. Most have either screened for the L1014F allele in West African countries (Martinez-Torres *et al*. 1998; Chandre *et al*. 1999b; Awolola *et al*. 2005; Coetzee *et al*. 2006), or the L1014S mutation in East Africa (Ranson *et al*. 2000; Kawada *et al*. 2011; Mawejje *et al*. 2012; Protopopoff *et al*. 2013). However some studies have screened for the presence of both resistance alleles in several parts of Africa (Stump *et al*. 2004; Etang *et al*. 2006; Pinto *et al*. 2006; Verhaeghen *et al*. 2006; Awolola *et al*. 2007; Moreno *et al*. 2008). Studies have demonstrated the presence of L1014S point mutation in West Africa (Djegbe *et al*. 2011) and L1014F mutation in East Africa (Kulkarni *et al*. 2006), indicating that the two mutations does not follow the previously described geographical distribution. Although several studies have been carried out in Tanzania to investigate the insecticide resistance status of the malaria vectors (Kulkarni *et al*. 2006, 2007; Kabula *et al*. 2012; Protopopoff *et al*. 2013), there has been no detailed information on the presence and the distribution of both *kdr* mutations in the country. This is the first such study designed to investigate the presence and the distribution of the two *kdr* mutations (L1014F and L1014S) in local population of *Anopheles gambiae s.l*. of Tanzania.

## Methods

### Study sites

**Figure 1:**
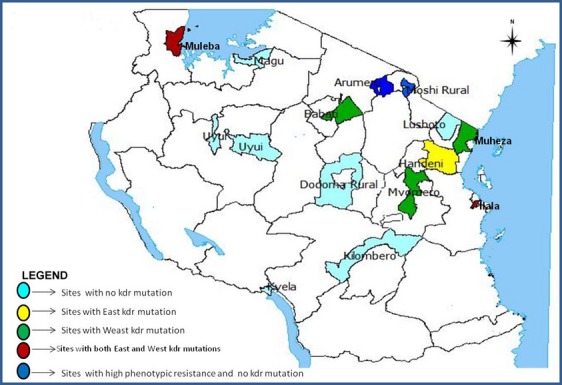
Map showing the geographical locations of the study sites and the distribution of East (L1014S) and West (L1014F) African knock-down resistance (*kdr*) mutations in *Anopheles gambiae s.l*. in Tanzania

The study was a follow-up to the main insecticide resistance survey carried in 2011. This was carried out in 10 sentinel districts across Tanzania mainland (Figure1), namely Muheza, Handeni, Lushoto, Arumeru, Uyui, Kyela, Ilala, Muleba, Kilombero and Mvomero. Additionally, this study used mosquitoes (for molecular analysis) collected in the main insecticide resistance survey from Moshi, Dodoma, Magu and Babati whose results have been reported elsewhere (Kabula *et al*. 2013). The study districts were chosen to encompass previously described WHO-recommended criteria (Kabula *et al*. 2012, 2013). The detailed characteristics of these study districts are described elsewhere (Kabula *et al*. 2012, 2013) and are summarised in Table1.

**Table 1:** Distribution of mosquitoes genotyped and characteristics of the study sites

Region	Site	*N*	(*N*) identified as *An. gambiae s.s*.	(*N*) identified as *An. arabiensis*	(*N*) Resistant to Lambdacyhalothrin	(*N*) Susceptible to Lambdacyhalothrin	Agricultural Insecticide Pressure (H/L) in the site
Tanga	Handeni	25	1	24	1	24	For crop protection (L)
Dar es Salaam	Ilala	25	9	16	3	22	For horticulture and Industrial pollution/effluents (H)
Manyara	Babati	25	12	13	0	25	For cereals plantations (H)
Tanga	Muheza	25	5	20	16	9	For crop protection (L)
Kagera	Muleba	25	21	4	15	10	For coffee protection (H)
Morogoro	Mvomero	25	0	25	0	25	For cereal & sugarcane protection (H)
Kilimanjaro	Moshi	25	0	25	25	0	For coffee, cereal & sugarcane protection (H)
Arusha	Arumeru	25	0	25	25	0	For floriculture and coffee plantations (H)
Mwanza	Magu	25	0	25	0	25	For cotton protection (H)
Tanga	Lushoto	25	25	0	0	25	For horticulture (H)
Morogoro	Kilombero	25	0	25	0	25	For cereal & sugarcane protection (H)
Tabora	Uyui	25	25	0	0	25	For tobacco protection (L)
Mbeya	Kyela	25	0	25	0	25	For cereal & cocoa protection (H)
Dodoma	Dodoma Rural	25	25	0	0	25	For crop protection (L)

(L/H): L – stands for low insecticide usage, H – stands for high insecticide usage; *N* = sample size.

### Mosquito sampling

Adult female Anopheles mosquitoes for susceptibility testing and molecular characterisation of insecticide resistance were collected by the indoor-resting catch technique (WHO 1975) in June–July 2011. Indoor-resting catches were carried out between 0600 and 0900 h in all locations. Freshly blood-fed and unfed female Anopheles mosquitoes were collected. Captured mosquitoes were collected in paper cups and transported to a field laboratory for morphological identification (Gillies & Coetzee 1987) and susceptibility testing (WHO 1998). They were fed with 10% sugar solution embedded in cotton wool pads during transportation. In Tabora, Lushoto and Muleba, the number of adult Anopheles mosquitoes was not sufficient for the susceptibility test; therefore, larvae were collected and reared to adults under standard laboratory conditions (WHO 1975).

### Insecticide susceptibility tests

The standard WHO susceptibility tests were conducted on field collected mosquitoes using test-kits and insecticide-impregnated filter papers supplied by the WHO (1975, 1998). Adult female Anopheles mosquitoes were exposed to 0.05% lambdacyhalothrin for 1 h. There were 4–9 replicates of 15–25 wild adult female mosquitoes per test. The controls were exposed to silicone oil impregnated paper. At this exposure time, the number of mosquitoes knocked down was recorded at 10, 15 20, 30, 40, 50 and at 60 min (WHO 1998, 2013). Mosquitoes were then transferred into the holding tube and fed on 10% (w/v) sugar solution for 24 h. Final mortality was scored after a 24-h holding. Insecticide susceptibility was classified according to the WHO criterion, which considers mortality of 98–100% and below 90% representative of susceptible and resistant populations, respectively, while the intermediates (90–97%) need further investigation (WHO 2013). Estimates for 50% knock-down time (KDT_50_) were assessed using log-probit analysis (Finney 1971).

### Mosquito identification

Mosquitoes were identified to species based on morphological characteristics (Gillies & Coetzee 1987) and stored individually over silica gel for molecular identification and detection of *kdr* variants. Surviving mosquitoes from susceptibility tests were killed by exposure to ether fumes or by freezing at -20°C prior to morphological identification and storage. All lambdacyhalothrin-resistant mosquitoes were picked from each sentinel site for molecular species identification and *kdr* analysis. Stored mosquito samples that were previously exposed to lambdacyhalothrin in the 2011 main insecticide resistance survey (Kabula *et al*. 2013) from Magu, Babati, Moshi and Dodoma were also used in this molecular analysis. In sites where the number of resistant mosquitoes was less than 25 or 0, *An. gambiae s.l*. were picked at random to make up the total number of 25 per site (Table1). Genomic DNA was extracted from the whole mosquito of a proportion of females using standard methods (Collins *et al*. 1987) and amplified using specific diagnostic primers for *An. gambiae s.l* (Collins *et al*. 1987; Scott *et al*. 1993).

### Detection of knock-down resistance (kdr) alleles in An. gambiae s.l

**Figure 2:**
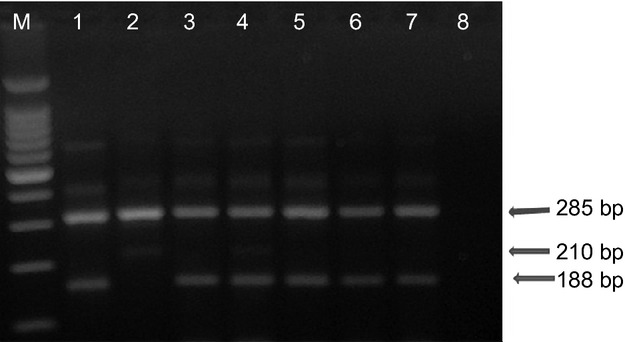
Gel electrophoresis of East African knock-down (L1014S) resistance assay. All successful reactions contain a band of 285 bp, a band of 210 bp indicates the susceptible (wild-type) allele and one of 188 bp the resistant allele. The first lane contains a 100-kb ladder marker, lane 1 is the control for the L1014S homozygous resistant, lane 2 is control for the L1014S homozygous susceptible. Lanes 3 and 5 are samples from Muleba. Lanes 4 and 6 are samples from Dar es Salaam (Ilala); lane 7, sample from Handeni; and lane 8, negative control.

**Figure 3:**
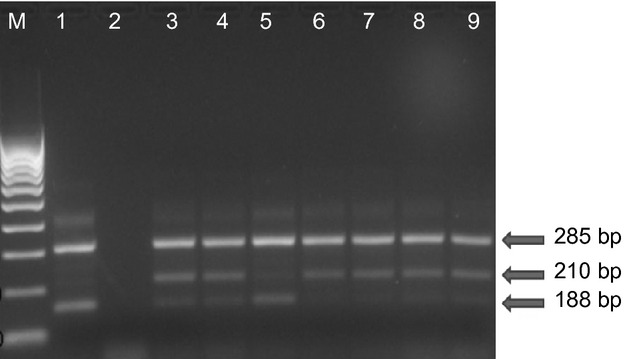
Gel electrophoresis of West African knock-down (L1014F) resistance assay. All successful reactions should contain a band of 285 bp, a band of 210 bp indicates the susceptible (wild-type) allele and one of 188 bp the resistant allele. The first lane contains a 100-kb ladder marker, lane 1 is the control for the L1014F homozygous resistant, lane 2 is a negative control, lanes 3–7 are samples from Muheza, Dar es Salaam (Ilala) and Muleba, respectively. Lanes 8 and 9 are samples from Babati (Magugu) and Mvomero respectively.

Mutations associated with knock-down resistance (i.e. L1014S and L1014F) to pyrethroids were assayed using the standard PCR assays (Martinez-Torres *et al*. 1998; Ranson *et al*. 2000). The PCR products were electrophoresed through 2% agarose gel with ethidium bromide stain and visualised under UV light. Successful reactions had a band of 285 bp. Additionally, there was a 210-bp band for wild-type susceptible and 188 bp for resistant allele (Figures2 and 3).

## Results

**Figure 4:**
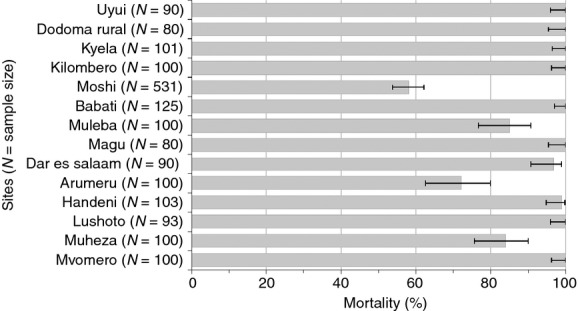
Mortality rates in field populations *of Anopheles gambiae s.l*. exposed to 0.05% lambdacyhalothrin for 60 min. 24-hmortalities <90% are indicative of resistance under WHO terminology and mortality of 90–97% indicates incipient resistance. *N *= number of mosquitoes exposed to lambdacyhalothrin. Mortality rates for Magu, Babati, Moshi and Dodoma were adapted from Kabula *et al*. (2013).

Mean mortality rates of An*. gambiae s.l*. 24 h post-exposure (Figure4) ranged from 72% to 100%. Full susceptibility to lambdacyhalothrin was observed in Mvomero, Lushoto, Handeni, Kilombero, Kyela and Uyui (mortality of 98–100%). Resistance to lambdacyhalothrin was recorded in Muheza, Arumeru and Muleba (mortality of 83.5%, 72%, and 85%, respectively), while Dar es Salaam recorded reduced susceptibility (mortality of 96.7%).

The median knock-down time (KDT_50_) of the wild mosquitoes ranged from 13.4 to 152.7 min. Highest KDT_50_ were recorded in Arumeru, Dar es Salaam and Muleba (KDT_50_ of 129, 42 and 39 min, respectively). The low KDT_50_ of 13.4 20.9, 21.2, 25, 27.7 and 31.9 min were recorded in Kyela, Muheza, Mvomero, Lushoto, Uyui, Kilombero and Handeni, respectively. The proportion of KDT_50_ of the wild populations to that of susceptible laboratory Kisumu mosquitoes known as resistance ratio (RR) was also calculated. Muleba, Dar es Salaam and Arumeru had the highest RRs. The KDT_50_ in these sites was between 2.6, 2.8 and 8.5 times than that of the control susceptible Kisumu strain, respectively.

A total of 1563 mosquitoes were morphologically identified as *An. gambiae s.l*. and tested for their susceptibility to lambdacyhalothrin. Of these, 350 (22% of the total morphologically identified mosquitoes) were identified to species level using PCR-based techniques. Of the 350, 123 (35.1%) were identified as *An. gambiae s.s*. and 227 (64.9%) as *An. arabiensis* (Table1). These 350 mosquitoes were also genotyped for *kdr-east* (L1014S) and *kdr-west* (L1014F) mutations. Of these, 341 were homozygous for the susceptible wild type and 9 were homozygous for L1014S genotype (Table2). When genotyped for L1014F, 317 were homozygous for the susceptible wild type and 33 were heterozygous (Table3). There was a significant difference in L1014S allele between lambdacyhalothrin-resistant and susceptible mosquitoes (Fisher exact *P *<* *0.000001). However, there was no significant difference in L1014F allele between lambdacyhalothrin-resistant and susceptible mosquitoes (χ^2^=0.68; *P *=* *0.409) (Table4). No L1014S allele was identified among lambdacyhalothrin susceptible (Table5).

**Table 2:** Distribution of *kdr*-East (L1014S) mutation in *An. gambiae s.s. and An arabiensis* mosquitoes

		*Anopheles gambiae s.s*.						*Anopheles arabiensis*				
		Genotype count			Allelic frequency			Genotype count			Allelic frequency	
Site	*N*	RR	RS	SS	R	S	*N*	RR	RS	SS	R	S
Handeni	1	0	0	1	0.000	1.000	24	1	0	23	0.042	0.958
Dar es Salaam	9	3	0	6	0.333	0.667	16	0	0	16	0.000	1.000
Babati	12	0	0	12	0.000	1.000	13	0	0	13	0.000	1.000
Muheza	5	0	0	5	0.000	1.000	20	0	0	20	0.000	1.000
Muleba	21	5	0	16	0.238	0.762	4	0	0	4	0.000	1.000
Mvomero	0	*	*	*	*	*	25	0	0	25	0.000	1.000
Moshi	0	*	*	*	*	*	25	0	0	25	0.000	1.000
Arumeru	0	*	*	*	*	*	25	0	0	25	0.000	1.000
Magu	0	*	*	*	*	*	25	0	0	25	0.000	1.000
Lushoto	25	0	0	25	0.000	1.000	0	*	*	*	*	*
Kilombero	0	*	*	*	*	*	25	0	0	25	0.000	1.000
Uyui	25	0	0	25	0.000	1.000	0	*	*	*	*	*
Kyela	0	*	*	*	*	*	25	0	0	25	0.000	1.000
Dodoma Rural	25	0	0	25	0.000	1.000	0	*	*	*	*	*

RR, RS and SS are three possible kdr genotypes, where R represents the resistant L1014S allele and S represents the susceptible wild-type allele.

* No member of a particular species were found in molecular identification, **that is,** all were identified as either *An. gambiae s.s*. or *An. arabiensis*.

**Table 3:** Distribution of *kdr*-west (L1014F) mutation in *An. gambiae s.s. and An arabiensis* mosquitoes

		*Anopheles gambiae s.s*.						*Anopheles arabiensis*				
		Genotype count			Allelic frequency			Genotype count			Allelic frequency	
Site	*N*	RR	RS	SS	R	S	*N*	RR	RS	SS	R	S
Handeni	1	0	0	1	0.000	1.000	24	0	0	24	0.000	1.000
Dar es Salaam	9	0	0	9	0.000	1.000	16	0	13	3	0.406	0.594
Babati	12	0	0	12	0.000	1.000	13	0	3	10	0.115	0.885
Muheza	5	0	0	5	0.000	1.000	20	0	8	12	0.200	0.800
Muleba	21	0	3	18	0.071	0.929	4	0	3	1	0.375	0.625
Mvomero	0	*	*	*	*	*	25	0	3	22	0.060	0.940
Moshi	0	*	*	*	*	*	25	0	0	25	0.000	1.000
Arumeru	0	*	*	*	*	*	25	0	0	25	0.000	1.000
Magu	0	*	*	*	*	*	25	0	0	25	0.000	1.000
Lushoto	25	0	0	25	0.000	1.000	0	*	*	*	*	1.000
Kilombero	0	*	*	*	*	*	25	0	0	25	0.000	1.000
Uyui	25	0	0	25	0.000	1.000	0	*	*	*	*	*
Kyela	0	*	*	*	*	*	25	0	0	25	0.000	1.000
Dodoma Rural	25	0	0	25	0.000	1.000	0	*	*	*	*	*

RR, RS and SS are three possible kdr genotypes, where R represents the resistant L1014S allele and S represents the susceptible wild-type allele.

* No member of a particular species were found in molecular identification, **that is,** all were identified as either *An. gambiae s.s*. or *An. arabiensis*.

**Table 4:** Number of mosquitoes with *kdr*-east (L1014S) and *kdr*-west (L1014F) mutation genotypes among surviving (resistant) and dead (susceptible) mosquitoes after exposure to lambdacyhalothrin

		*kdr*-east genotype				*kdr*-west genotype			
	*n*	RR	RS	SS	Statistics	RR	RS	SS	Statistics
Resistants (surviving)	85	9	0	76	Fisher's exact test *P *<* *0.000001	0	10	75	χ^2^ = 0.68; *P *=* *0.409
Susceptibles (dead)	265	0	0	265		0	23	242	

RR, RS and SS are three possible kdr genotypes, where R represents the resistant L1014S or L1014F allele and S represents the susceptible wild-type allele.

**Table 5:** Number of mosquitoes with *kdr-east* (L1014S) and *kdr-west* (L1014F) genotypes among *An. gambiae* s.s. and *An. arabiensis*

		*kdr*-east genotype				*kdr*-west genotype			
	*n*	RR	RS	SS	Statistics	RR	RS	SS	Statistics
*An. gambiae s.s*.	123	8	0	115	χ^2^ = 23.41; *P *<* *0.0001	0	3	120	χ^2^ = 11.21; *P *=* *0.0008
*An. arabiensis*	227	1	0	226		0	30	197	

RR, RS and SS are three possible kdr genotypes, where R represents the resistant L1014S or L1014F allele and S represents the susceptible wild-type allele.

The distribution of L1014S and L1014F mutations in *An. gambiae s.s*. and *An. arabiensis* in different parts of the country is shown in Tables2 and 3 and in Figure1. The L1014S mutation was detected in both *An. gambiae s.s*. and *An. arabiensis*. The L1014S mutation was found at the allelic frequency of 33.3% in Dar es Salaam (95% CI: 16–56%) and 23.8% in Muleba (95% CI: 13–38.5%) in *An. gambiae s.s*.; and 4.2% (95% CI: 1.1–13.9%) of *An. arabiensis* from Handeni. Similary, the L1014F point mutation was detected in both *An. gambiae s.s*. and *An. arabiensis*. The L1014F mutation was found in *An. gambiae s.s*. from Muleba at the allelic frequency of 7.1% (95% CI: 2.5–19%). This L1014F mutation was found in *An. arabiensis* at the allelic frequency of 40.6% in Dar es Salaam (95% CI:25.5–57.7%), 11.5% in Babati (95% CI:4–28.9%), 20% in Muheza (95% CI:10.5–34.8%), 37.5% in Muleba (95% CI:13.7–69.4%) and 6% in Mvomero (95% CI:2–16.2%). The L1014S and L1014F mutations occurred together in Muleba and Dar es Salaam (Figure1). Although the two kdr mutations appeared in both *An. gambiae s.s*. and *An. arabiensis,* the L1014F was much associated with *An. arabiensis* (χ^2^ = 11.21; *P *=* *0.0008) while the L1014S was associated with *An. gambiae s.s*. (χ^2^ = 23.41; *P *<* *0.0001) (Table5).

## Discussion

Results from this study continued to demonstrate that the field population of *An. gambiae s.l*. are resistant to lambdacyhalothrin. Resistance of these malaria vectors to pyrethroids has previously been reported in Tanzania (Kabula *et al*. 2012, 2013; Protopopoff *et al*. 2013). The persistence of such resistance could be due the pressure created by the cumulative effect of insecticides used in malaria vector control and agriculture (Kabula *et al*. 2012, 2013). This study also reports the countrywide distribution of kdr mutations (L1014S and L1014F) in members of *An. gambiae s.l*. It reports the presence and wide distribution of the L1014S mutation in *An. gambiae s.s*. and *An. arabiensis* in Tanzania. It also further confirms the presence of L1014F point mutation in *An. gambiae s.s*. and *An. arabiensis*. The L1014S and L1014F mutations were detected in both *An. gambiae s.s*. and *An. arabiensis*. However, L1014S mutation was frequently found in *An. gambiae s.s*. while L1014F was frequently found in *An. arabiensis*. Presence of L1014F mutation at very low frequency in *An. arabiensis* had previously been reported in the country (Kulkarni *et al*. 2006) and in the neighbouring Kenya and Uganda (Stump *et al*. 2004; Kawada *et al*. 2011; Mawejje *et al*. 2012). The occurrence of both mutations in *An. gambiae s.s*. and *An. arabiensis* in this study may indicate that these mosquitoes have similar exposure to the sources which create selection pressure for knock-down resistance. The difference in their frequency of these mutations in the two members of *An. gambiae s.l*. may, however, be related to a different origin of the mutations in the two populations or linked to different ecological or behavioural characters between *An. gambiae s.s*. and *An. arabiensis* (Stump *et al*. 2004).

The L1014S mutation was detected in *An. gambiae s.s*. from Dar es Salaam (allelic frequency of 33%) and Muleba (allelic frequency of 24%) and in *An. arabiensis* from Handeni (allelic frequency of 4%). The L1014F mutation was found in *An. gambiae s.s*. from Muleba (allelic frequency of 7%) and in *Anopheles arabiensis* from Babati, Dar es Salaam, Muheza, Muleba and Mvomero. The L1014S and L1014F mutations co-occurred in Muleba and Dar es Salaam. The high frequency of kdr mutations in Muleba district, also previously reported (Protopopoff *et al*. 2013), may be a response to selection by recurrent IRS with lambdacyhalothrin since 2007, increased use of permethrin LLINs in association with the extensive usage of pesticides in coffee plantations. However, kdr has been reported in some areas with no IRS pressure in Burundi (Protopopoff *et al*. 2008) – which explains the occurrence of kdr in Handeni. Low insecticide usage in Handeni for agriculture may also play a role in the occurrence of kdr mutation. High frequency of kdr mutation in Dar es Salaam may be attributed to increased selection pressure resulting from industrial waste/pollutants, high LLINs use (Kabula *et al*. 2013) and extensive local use of insecticides for fumigation and agricultural (mainly horticulture) purposes. The high kdr frequency in Dar es Salaam is supported by the previous report of high level of DDT resistance(Kabula *et al*. 2012). Occurrence of kdr mutations in Muheza, Babati and Mvomero may be attributed to the high LLINs use and use of pyrethroids in agriculture.

Selection of knock-down resistance has been attributed mainly to the use of DDT and pyrethroids in agriculture and public health (Elissa *et al*. 1993; Stump *et al*. 2004). For example, the use of pyrethroids in malaria vector control interventions such as ITNs and IRS is known to create the selection of kdr alleles (Stump *et al*. 2004; Protopopoff *et al*. 2013). Similarly, domestic use of insecticides (e.g. fumigation) may play an important role in selection of knock-down resistance (Elissa *et al*. 1993), and this may be the case for urban settings such as Dar es Salaam.

The L1014S allele occurred significantly more often in lambdacyhalothrin phenotypically resistant-selected samples than in susceptible ones. Apart from the association found in this study, some sites which previously reported pyrethroid and DDT resistance (Kabula *et al*. 2012, 2013; Protopopoff *et al*. 2013) were found with kdr mutations (e.g. Muheza, Muleba). Such resistance to pyrethroids and DDT in *An. gambiae* is known to associate closely with both L1014S and L1014F mutations (Williamson *et al*. 1996; Martinez-Torres *et al*. 1998; Ranson *et al*. 2000, 2004; Reimer *et al*. 2008). However, the association was not found in the case of L1014F mutation and the pyrethroid-resistant phenotypes. Similarly, this study could not establish such associations in some sites (e.g. Mvomero and Babati) where *kdr* mutations were recorded without obvious phenotypic resistance to pyrethroids being observed. The absence of pyrethroid phenotypic resistance in Mvomero and Babati may be explained by the recessiveness of the kdr allele. Henceforth, the occurrence of the genes in heterozygous recessive form leads to their appearance at low frequencies in these two sites. This might explain the absence of phenotypic resistance to pyrethroids, as the conventional bioassay methods that measure phenotypic resistance cannot detect the heterozygous proportion of the population (Chandre *et al*. 2000). However, models of insecticide resistance show rapid increase in the frequency of resistance, especially when the frequency reaches levels as low as 0.1%, resulting in control failure (Roush & McKenzie 1987). Conversely, the presence of *kdr* mutation in Babati is strongly supported by KDT_50_ for lambdacyhalothrin. High values of KDT_50_ in the field mosquitoes gives early indication of the presence of kdr mutation (Chandre *et al*. 2000). A significant increase in knock-down time may be observed in some mosquito populations before any decrease in mortality, suggesting that knock-down time could also be a good indicator for the early detection of pyrethroid resistance (Chandre *et al*. 2000).

Mosquitoes from Moshi and Arumeru did not have *kdr* mutations despite having high levels of phenotypic pyrethroid resistance (Kabula *et al*. 2013). This suggests that other mechanisms are responsible for the observed phenotypic resistance in these sites. Possibly the main mechanisms involved in these sites might be biochemical resistance which had previously been reported in Moshi (Matowo *et al*. 2010). Even in the areas where the kdr mutations were found, the presence of other mechanisms cannot be ruled out. Both target-site insensitivity and metabolic resistance have been found in *An. gambiae* (Vulule *et al*. 1999; Stump *et al*. 2004; Mitchell *et al*. 2012). Therefore, there is a need to further investigate the presence and distribution of cytochrome P450-based metabolic resistance mechanisms in malaria vectors. Such information will help to explain the mechanism(s) of resistance responsible for the observed or even suspected resistance and thus facilitate planning for appropriate insecticide resistance management.

This study reports the countrywide distribution of L1014S and L1014F kdr mutations among members of *An. gambiae s.l.,* and further confirms the presence of a typically West African L1014F kdr mutation in Tanzania. Therefore, we re-emphasise the need to test for both kdr mutations regardless of geographical location (Kulkarni *et al*. 2006). Sequencing analysis is required to provide further insights on the phylogenetic relations of the L1014F alleles found in East and West Africa. We also reported the presence and wide distribution of the L1014S mutation in *An. gambiae s.s*. and *An. arabiensis* in Tanzania. The presence of these kdr mutations in the mosquito populations has since been used as predictor for their resistance to DDT and pyrethroids (Ranson *et al*. 2004; Reimer *et al*. 2008). These findings coupled with previous reports on insecticide resistance in the country (Kabula *et al*. 2013; Protopopoff *et al*. 2013) suggest that pyrethroid resistance is a widespread phenomenon among our malaria vector populations.

The implications of high kdr frequency on the malaria vector control interventions such as ITNs and IRS are uncertain. However, studies in Benin showed some reduced effectiveness of LLINs and IRS in areas where *An. gambiae* have high kdr frequency (N'guessan *et al*. 2007; Asidi *et al*. 2012). Thus, the potential operational impact of insecticide resistance on the effectiveness of vector control interventions such as ITNs and IRS needs to be properly evaluated. Meanwhile, periodic monitoring of the frequency of both L1014S and L1014F mutations and phenotypic pyrethroid resistance in *An. gambiae s.l*. is essential for the rational and effective control of these vectors.
